# Dental problems and chronic diseases in mentally ill homeless adults: a cross-sectional study

**DOI:** 10.1186/s12889-020-08499-7

**Published:** 2020-03-30

**Authors:** Cilia Mejia-Lancheros, James Lachaud, Rosane Nisenbaum, Andrea Wang, Vicky Stergiopoulos, Stephen W. Hwang, Patricia O’Campo

**Affiliations:** 1 MAP Centre for Urban Health Solutions, Li Ka Shing Knowledge Institute, St Michael’s Hospital, Unity Health Toronto, 30 Bond St, Toronto, ON M5B 1W8 Canada; 2 Applied Health Research Centre, Li Ka Shing Knowledge Institute, St Michael’s Hospital, Toronto, Canada; 3 Centre for Addiction and Mental Health, Toronto, Canada; 4 Department of Psychiatry, University of Toronto, 250 College Street, Toronto, Canada; 5 Division of General Internal Medicine, Department of Medicine, University of Toronto, Toronto, Canada; 6 Dalla Lana School of Public Health, University of Toronto, Toronto, Canada

**Keywords:** Oral health, Mouth diseases, Chronic diseases, Multiple chronic conditions, Inflammation, Homeless persons, Dental health services, Preventive health service

## Abstract

**Background:**

Dental problems (DPs) and physical chronic diseases (CDs) are highly prevalent and incident in people with low socioeconomic status such as homeless individuals. Yet, evidence on the association between DPs and physical CDs in this population is limited. In the present study, we assessed the association between DPs and type and number of CDs in individuals experienced chronic homelessness and serious mental health problems.

**Methods:**

We analyzed cross-sectional baseline data from 575 homeless adults with serious mental health problems participating in the Toronto site of the At Home/Chez Soi randomized controlled trial. Chronic DPs (lasting at least 6 months) were the primary exposure variable. Presence of self-reported CDs, including heart disease, effect of stroke, hypertension, diabetes, asthma, chronic bronchitis/emphysema, stomach or intestinal ulcer, inflammatory bowel disease, migraine, thyroid problems, arthritis, kidney/bladder problems, liver disease (other than hepatitis), and iron-deficiency anemia, were the primary outcomes. The total number of CDs was also analyzed as a secondary outcome.

Logistic regression models were used to assess the association between DPs with each of the studied CDs, and negative binomial regression was used to test the association between DPs with the number of CDs.

**Results:**

In our 575 homeless participants (68.5% males) with mean age 40.3 (11.8) years, a high proportion had DPs (42.5%). The presence of DPs was positively associated with heart disease (adjusted odds ratio (AOR):4.19,1.67–10.52), diabetes (AOR:2.17,1.13–4.17), chronic bronchitis (AOR:2.34,1.28–4.29), stomach or intestinal ulcer (AOR:3.48,1.80–6.73), inflammatory bowel disease (AOR:2.52,1.38–4.60), migraine (AOR:1.80,1.20–2.72), arthritis (AOR:2.71,1.71–4.29), kidney/bladder problems (AOR:2.43,1.30–4.54), and iron-deficiency anemia (AOR:3.28,1.90–5.65). DPs were also associated with a higher number of CDs (IRR: 1.62,1.38–1.90).

**Conclusion:**

Dental health problems in homeless individuals with serious mental disorders are associated with several CDs. Dental care should be better integrated into existing social and health programs serving this population to improve their overall health status.

The AH/CS study is registered with the International Standard Randomized Control Trial Number Register (ISRCTN42520374).

## Background

Oral hygiene is considered an important indicator of general health [1]. However, the high prevalence of dental problems (DPs), such as periodontitis, tooth decay and tooth loss, remain a public health concern worldwide. In 2017, 3.58 billion people were estimated to have DPs, with dental caries of permanent teeth being the most prevalent condition afflicting about 2.44 billion people [2]. DPs vary by socioeconomic position (SEP) with those with lower incomes or education [3] or individuals who have no fixed address having more DPs and other poor health conditions such as physical chronic diseases (CDs) [1, 4, 5].

DPs have been found to be associated with various CDs, including cardiovascular diseases (CVD) [6–8], metabolic syndrome [9], diabetes [10], lung [11] and kidney problems [12] in the general population. Several risk factors have been found to be associated with both DPs and CDs, including smoking, alcohol use, and having an unhealthy diet [1, 13]. Although these shared behavioral risk factors could partially explain the link between DPs with CDs, the underlying social and structural mechanisms remain unclear. Furthermore, much of the existing literature on DPs and CDs has been conducted in the general population rather than in groups of lower SEP or individuals who are precariously housed.

Individuals who are homeless (those without a fixed, regular, and adequate night-time residence) had higher mortality rates and experience the dual burdens of DPs, CDs compared to the general population [4, 5, 14] due to structural and system-level barriers (e.g., lack of affordable housing, shrinking safety nets, health care systems models, health services affordability, lack of accessibility to health and social services) [15–19], social exclusion [18]. People experiencing homelessness frequently suffer serious mental disorders (e.g., psychotic disorders, mood and personality disorders, substance and alcohol use disorders) [20, 21] and modifiable risk factors (e.g., diet, smoking) [22, 23], which also contribute to poor health profiles and premature mortality [5, 21]. Discrimination and stigma, and power imbalance between health care providers and marginalized clients also act as critical barriers to seeking, accessing, and obtaining the necessary and appropriate health care to meet the complex health and psychosocial needs of individuals experiencing homelessness [16–18, 24, 25]. Few studies have assessed the relationship between DPs with CDs among individuals socially excluded populations (i.e. Homeless people, prisoners, sex workers). The evidence is even scanter in individuals faced both homelessness and severe mental disorders; yet, DPs have been found to be associated with some mental health problem such as depression in homeless people [26]. Thus, a better understanding of the association between oral problems and CDs in homeless individuals with serious mental health problems would help inform the importance of integrating oral health within the social and health programs serving this population [18], as well as to scale structural policy and interventions to address the social causes (“the cause of cause”) of morbidity and health inequities among homeless people [27, 28].

## Methods

The present study, carried out within the Canadian socioeconomic and health system context, seeks to investigate the following research questions: (1) Is there an association between dental health problems and selected CDs (included CVD, diabetes; lung, gastrointestinal, and liver diseases; migraine, thyroid, arthritis, and kidney/bladder problems; and iron-deficiency anemia) in homeless people living with serious mental illness? (2) Is the number of CDs higher among those reporting chronic DPs compared with those not reporting chronic DPs?

### Study design and population

The present cross-sectional study is embedded in the Toronto site of the At Home/Chez Soi (AH/CS) demonstration project, a large Canadian multi-site randomized trial of a Housing First intervention for homeless individuals conducted in five cities across Canada [29]. A detailed description of the AH/CS study design, population, intervention and instruments has been published elsewhere [29]. Briefly, the AH/CS study recruited and collected baseline data on 575 people between October 2009–July 2011 who were homeless and had serious mental health problems living in Toronto. The AH/CS participants were followed on average 6 years between 2009 and 2017 [29]. In the present study, we used the AH/CS Toronto participants’ baseline data collected between October 2009–July 2011.

The Toronto AH/CS study has received approval by the St. Michael’s Hospital Research Ethics Board (Canada). The AH/CS study is also registered with the International Standard Randomized Control Trial Number Register (ISRCTN42520374).

### Study measures

Information related to study participants’ demographics, socioeconomics, mental and physical health, substance use, visits to health providers, housing status, community integration and recovery were collected using validated questionnaires and scales [29].

#### Main exposure

In the present analysis, chronic DPs (present or absent) was considered the primary exposure variable. To capture the severity and chronicity of their DPs, AH/CS participants were asked at baseline to report DPs that have lasted for at least 6 months.

#### Outcomes

The primary outcomes were the CDs (present or absent) reported at baseline, including heart disease, effect of stroke, hypertension, diabetes, asthma, chronic bronchitis/emphysema, stomach or intestinal ulcer, inflammatory bowel disease (Crohn’s disease, colitis) (IBD), migraine, thyroid problems, arthritis, kidney/bladder problems, liver disease (other than hepatitis), and iron-deficiency anemia. Except for hypertension (see below), information for the CDs was collected using the reporting format for common physical health conditions used by Statistics Canada surveys (the Canadian Community Health Survey and the National Population Health Survey) and adapted for this study. Participants were asked to report those physical conditions that lasted at least 6 months prior to the date of the AH/CS baseline interview. Hypertension was indicated if participants reported high blood pressure at AH/CS baseline or if their average systolic blood pressure was ≥135 mmHg or diastolic blood pressure was ≥85 mmHg [30]. The average was based on three repeated measures taken at the AH/CS baseline. Blood pressures were taken from the participant’s right arm using the automatic LifeSourceUA-767 plus monitor. The number of CDs presented (among the previous CDs) was considered an indicator of cumulative comorbidities and defined as the secondary outcome.

#### Covariates

Based on their epidemiological and clinical relationship with the DPs the following confounding factors measured at baseline were considered for the analysis adjustment. ***Demographics****:* Gender was categorized as male and female. Transsexual (*n* = 1) and transgender (*n* = 9) participants were included in the female category as these subgroups were too small for analysis as independent categories. Age was determined at baseline. ***Self-Ethno-racial and cultural identity*:** Categorized as white, black, and other ethno-racial group was considered among the adjustment variables as it could be a potential genetic and behavioral confounder. **Socioe*conomic factors:*** education level (attended some middle/high school, completed high school, and attended/completed college, trade school, or university) was considered an indicator of SEP. Lifetime length of homelessness (< 3 years/≥3 years**)** was included as indicator of exposure to accumulated adverse socioeconomic conditions and exclusion which can contribute to poor health including DPs and CDs [31]. **Level of needs for mental health services**: High need and moderate needs was considered because all participants had mental disorders, which according to their severity can have a detrimental influence on physical health if their needs for health care and social services are not appropriately met.

***Risk factors***: Smoking, drug abuse or dependence, and alcohol abuse or dependence (measured with the Mini-International Neuropsychiatric Interview), and BMI (kg/m^2^).

### Statistical analysis

The description of the study participants’ characteristics, including identifying those with and without DPs (Additional file 1, Table 1) were generating using frequencies, percentages, and means (±SD). Comparison of the baseline characteristics across DPs was assessed using the Chi-squared test for categorical variables and t-test for continuous variables (Table 1). There were some missing values (less than 18%) for DPs, CDs and covariates (Additional file 1, Table 1) due to non-response, item nonresponse, or low interviewer confidence rate in the participant’s answers. Considering these factors, the missingness of our data was at random. To minimize potential selection analysis bias, increase the power and the precision of the estimates, and reduce the potential lack of robustness of the complete case analysis [32–34], we used multiple imputation via Fully Conditional Specification approach to handle the missing data [35]. Although less than 20% of the data was missing, we imputed 100 datasets (10,000 interactions) to reduce the Monte Carlo error, to improve the efficiency of the parameters estimated and to prevent the power falloff [35, 36], using Stata 15.0 (command mi impute chained). In the imputed models, we included the DPs, each of the analysed CDs, and all the covariates used in the analysis, including those without missing data (age, gender, drug abuse or dependence, alcohol abuse or dependence, level of needs for mental health services). Table 2 in the Additional file 1, summarizes the variables, which have their values observed, imputed, and completed, as well as the statistical model specification (e.g., logistic regression, predictive mean matching, multinomial logistic regression) used for imputed each variable within the imputed model. The comparison of the imputed, completed and observed data for each of the imputed variables using the multiple imputation diagnostic plots, Kolmogorov–Smirnov test, and tables of proportions [37] showed good appropriateness of the imputation model and imputed values (See Additional file 1, Figure 1 and Tables 3 to 21) and therefore of the obtained findings. The results regarding the associations between the DPs and CDs presented in the present study, are those pooled from the 100 imputed datasets according to “Rubin’s rules” [38].

**Table 1:** Bivariate description of the baseline characteristics across chronic dental problems (DPs) in the AH/CS participants, Toronto Site (Observed data)

Characteristics at baseline	*N* = 546	No DPs (*n* = 314) (57.51%)	Yes DPs^a^ (*n* = 232) (42.49%)	*P*-value
**Demographics and socioeconomics**				
**Gender**^**b**^	**546**			
Male	373	53.62	46.38	0.007
Female	173	65.90	34.10	
**Age (years) at baseline**	**546**	38.11 (11.82)	43.47 (10.99)	0.000^t^
**Self-identified ethno-racial and cultural identity**^**c**^	**546**			
White	194	54.12	45.88	0.010
Black	187	66.31	33.69	0.000^¥^
Other	165	51.52	48.48	
**Lifetime length of homelessness**	**534**			
≤ 3 years	248	68.95	31.05	0.000
> 3 years	286	48.25	51.75	
**Education**	**546**			
Attended some middle/high school	260	55.00	45.00	0.474
Completed high school	104	61.54	38.46	0.365^¥^
Attended/completed college, trade, school, or university	182	58.79	41.21	
**Level of needs for mental and social services**	**546**			
Moderate needs	366	56.83	43.17	0.647
High needs	180	58.89	41.11	
**Lifestyles and risk factors**				
**Smoking**	**544**			
No	146	72.60	27.40	0.000
Yes (daily or occasionally)	398	52.26	47.74	
**Drug abuse or dependence**	**546**			
No	288	65.28	34.72	0.000
Yes	258	48.84	51.16	
**Alcohol abuse or dependence**	**546**			
No	310	61.61	38.39	0.026
Yes	236	52.12	47.88	
**BMI (kg/m**^**2**^**)**	**468**	27.05 (6.47)	25.69 (5.55)	0.019^t^

^a^Yes dental problem refers to self-reported of having DP lasted at least 6 months at AH/C-S baseline

^b^Female includes individuals identified as female and those as transsexual (*n* = 1) or transgender (*n* = 9) at AH/CS study's baseline

^c^The black group includes African-black, Caribbean region-black, and Canadian-black. The white group includes European-white and Canadian-white. Other ethno-racial group includes Indigenous, East Asian, South Asian, South-Eastern Asian, Latin American, Indians, and Caribbean, Middle Eastern, mixed

t = t-test, ¥= p-trend

Logistic regression models were used to test the association (odds ratio and 95% confidence interval) between the baseline DPs and each of the studied CDs. Negative binomial regression (due to over-dispersion) were used to assess the association (incident rate ratio and 95% confidence interval) of DPs and the number of CDs. Variables were added to the model according to the following steps. A univariate association between having DPs and each of the studied CDs and the number of CDs was initially assessed (Model 1). For those CDs with events with more than 30 observations, the association were adjusted for the demographic characteristics (Model 2), followed by adding ethno-racial characteristics (Model 3), socioeconomic factors (Model 4), level of need (Model 5), and risk factors (Model 6). For those CDs with less than 30 observations (i.e., heart disease, stroke, thyroid problems, and liver disease), the crude associations were adjusted for age, ethno-racial characteristics, and level of need for mental health services, to ensure essential confounding control [39], to avoid power decline to detect potential associations between the exposure and the outcomes, and to increase precision of the estimations.

The area under the receiver operating characteristic curve (AUC) was used to assess the fit of the models. All analyses were performed using Stata software, version15.0 and statistical tests were two-sided. *P*-values less than 0.05 were defined as statistical significance.

## Results

A high proportion of participants, 42.49%, reported DPs at baseline. Hypertension (41.2%), migraine (29.8%), arthritis (24.6%), asthma (20.3%) were the more prevalent CDs, whilst heart disease (5.1%), stroke (4.0%), thyroid problems (3.58%), and liver disease (3.38%) were the less prevalent CDs. The average number of CDs was 2.0 (SD: 1.8) (Additional file 1, Table 1).

### Description of the participants’ characteristics across DPs

After comparing participants with chronic DPs and those without DPs, it was found that being male, older and identifying as other ethno-racial groups was related to having a higher percentage of DPs (Table 1). Similarly, participants with a longer length of lifetime homelessness, those who were smokers and misused drugs and alcohol had DPs more frequently (Table 1). Conversely, having higher BMI was related to a lower proportion of DPs (Table 1).

### Association between DPs and CDs

**Fig. 1:**
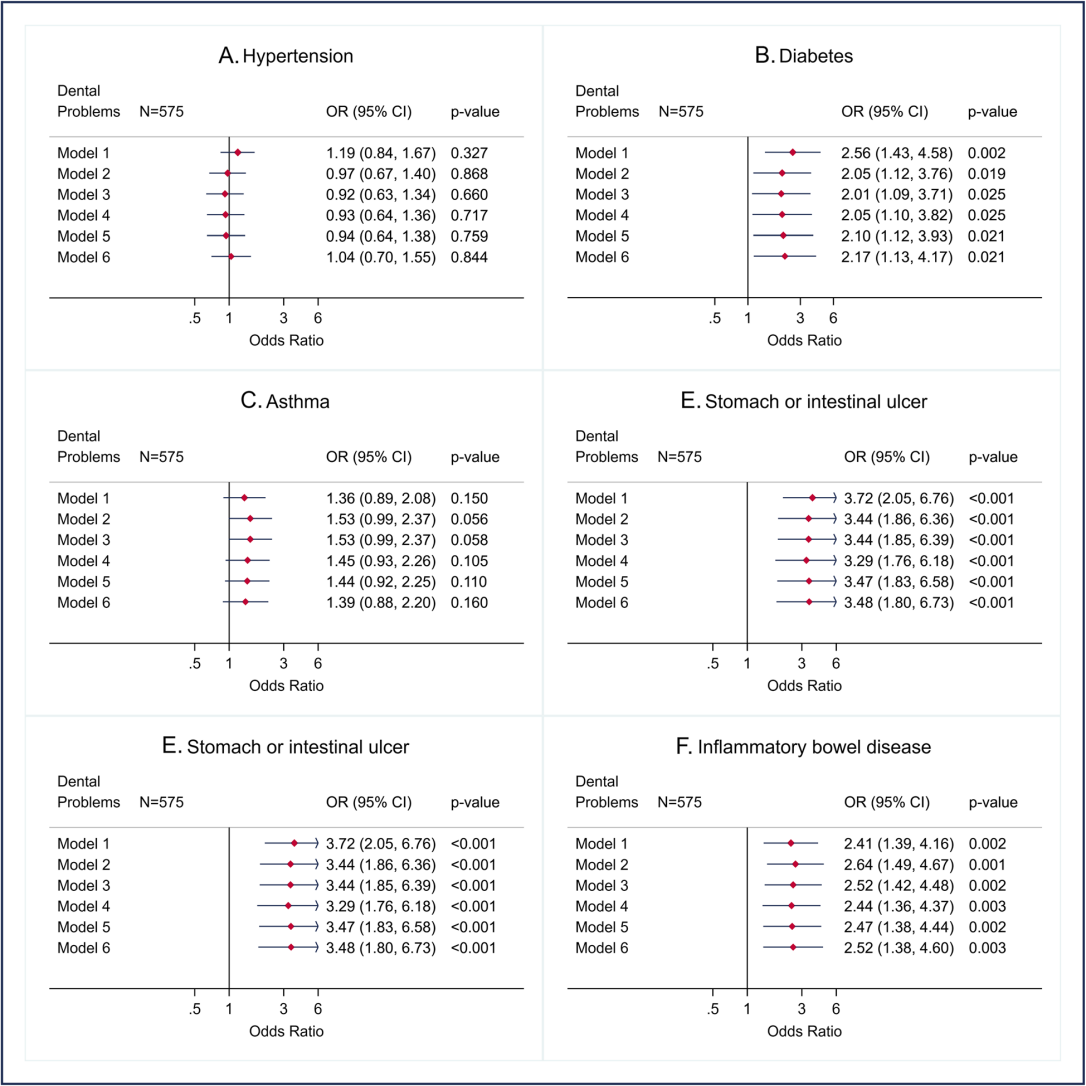
Crude and adjusted odds ratio (OR) (CI 95% and *p*-value) for hypertension, diabetes, asthma, chronic bronchitis/emphysema, stomach or intestinal ulcer, and inflammatory bowel disease according to dental problems in AH/CS participants, Toronto Site (imputed data). Model 1. Crude OR. Model 2. OR adjusted for gender and age. Model 3. OR adjusted for gender, age, and ethno-racial group. Model 4. OR adjusted for gender, age, and ethno-racial group, lifetime of homelessness, and education. Model 5. OR adjusted for gender, age, and ethno-racial group, lifetime of homelessness, education, and level of needs. Model 6. OR adjusted for gender, age, and ethno-racial group, lifetime of homelessness, education, level of needs, smoking, drug abuse or dependence, alcohol abuse, and BMI. Fit of the full model (Model 6): Hypertension: Model 6, AUC = 0.72. Diabetes: Model 6, AUC = 0.73. Asthma: AUC = 0.63. Chronic bronchitis/emphysema: model 6, AUC = 0.78. Stomach or intestinal ulcer: Model 6, AUC = 0.74. Inflammatory bowel disease, AUC = 0.73

**Fig. 2:**
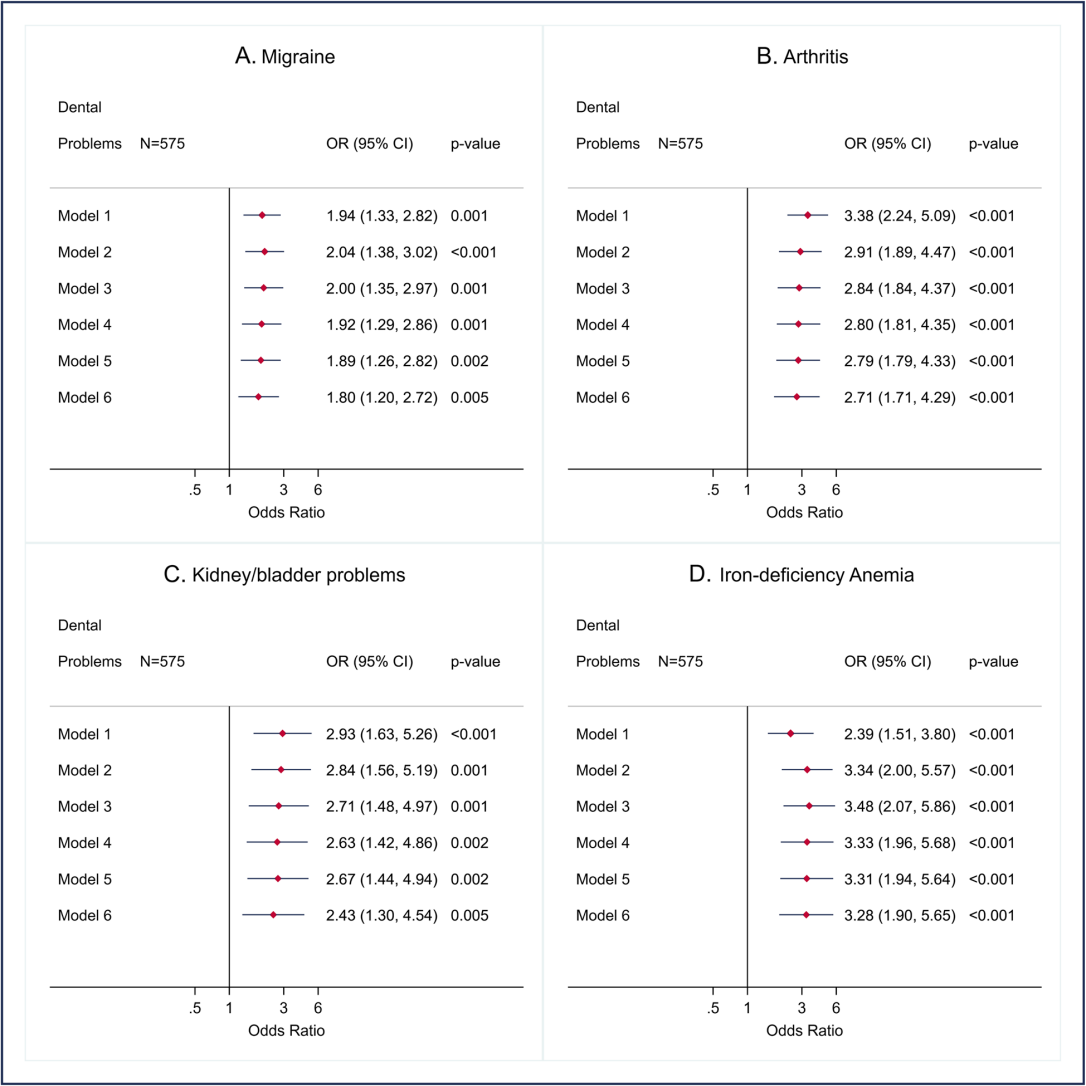
Crude and adjusted odds ratio (OR) (CI 95% and p-value) for migraine, arthritis, kidney/bladder problem, and iron-deficiency anemia according to dental problems in AH/CS participants, Toronto Site (imputed data). Model 1. Crude OR. Model 2. OR adjusted for gender and age. Model 3. OR adjusted for gender, age, and ethno-racial group. Model 4. OR adjusted for gender, age, and ethno-racial group, lifetime of homelessness, and education. Model 5. OR adjusted for gender, age, and ethno-racial group, lifetime of homelessness, education, and level of needs. Model 6. OR adjusted for gender, age, and ethno-racial group, lifetime of homelessness, education, level of needs, smoking, drug abuse or dependence, alcohol abuse, and BMI. Fit of the full model (Model 6): Migraine: Model 6, AUC = 0.65. Arthritis: Model 6, AUC = 0.75. Kidney/bladder problem: Model 6, AUC = 0.72. Iron-deficiency Anemia: model 6, AUC = 0.7

**Fig. 3:**
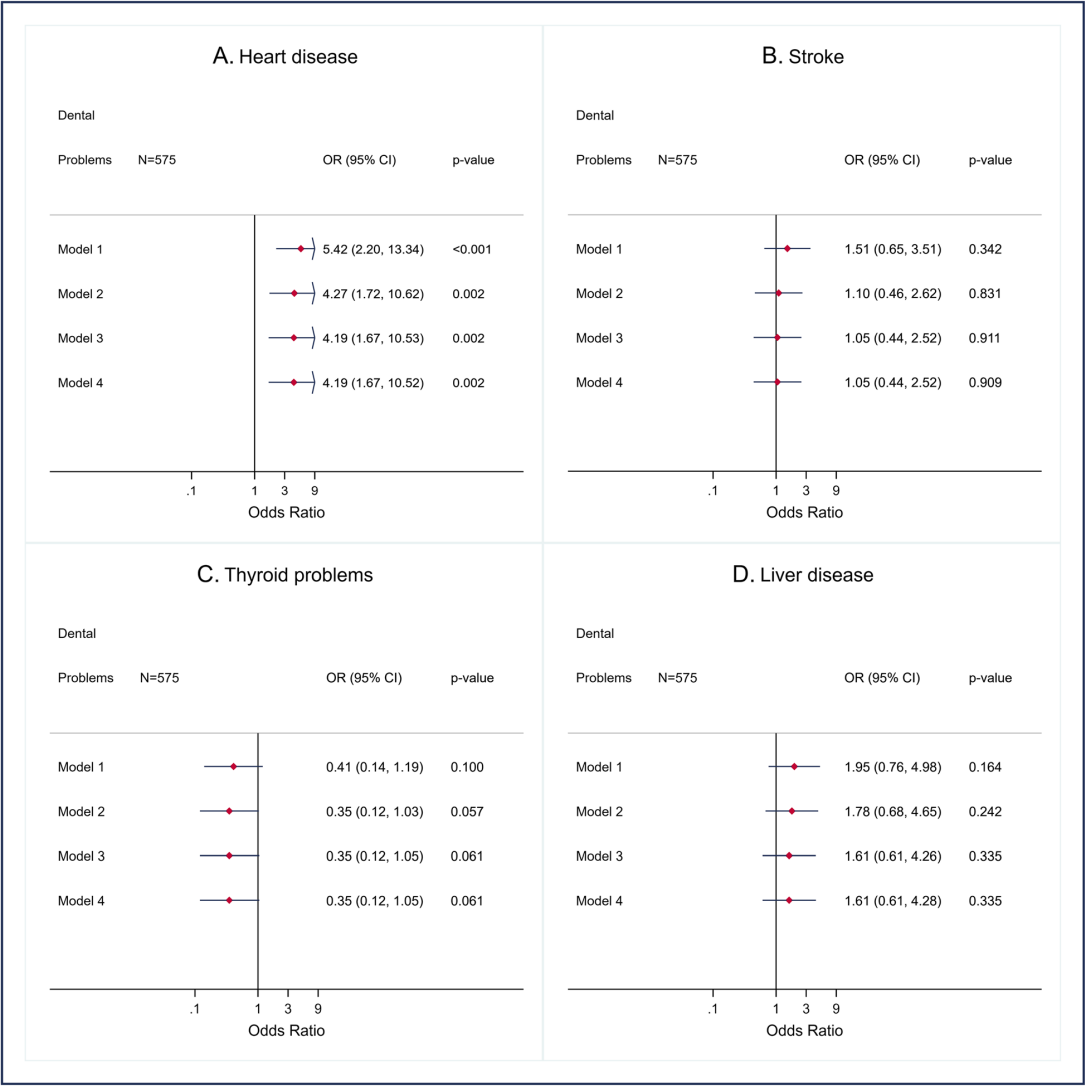
Crude and adjusted odds ratio (OR), (95% CI and p-value) for heart disease, stroke, thyroid problems, and liver disease according to dental problems in AH/CS participants, Toronto Site (imputed data). Model 1. Crude OR. Model 2. OR adjusted for age. Model 3. OR adjusted for age, and ethno-racial group. Model 4. OR adjusted for age, and ethno-racial group, and level of needs. Fit of the full model (Model 6): Heart disease: Model 4, AUC = 0.83. Stroke: Model 4, AUC = 0.75. Thyroid problem: Model 4, AUC = 0.66. Liver disease: model 4, AUC = 0.69

**Fig. 4:**
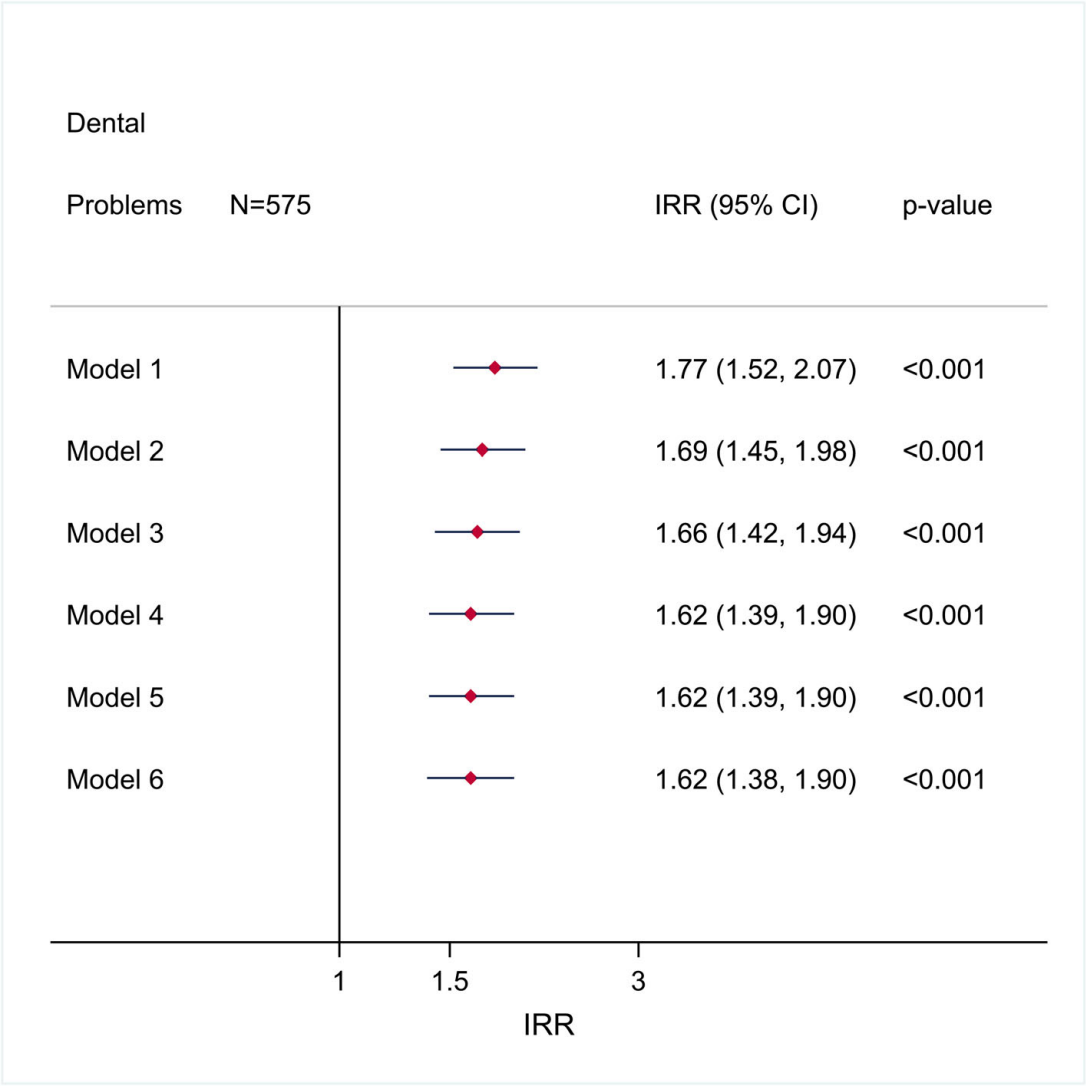
Crude and adjusted incident rate ratios (IRR) (CI 95% and p-value), for number of chronic diseases according to dental problems in AH/CS participants, Toronto Site (imputed data). Model 1. Crude IRR. Model 2. IRR adjusted for gender and age. Model 3. IRR adjusted for gender, age, and ethno-racial group. Model 4. IRR adjusted for gender, age, and ethno-racial group, lifetime of homelessness, and education. Model 5. IRR adjusted for gender, age, and ethno-racial group, lifetime homelessness education, and level of needs. Model 6. IRR adjusted for gender, age, and ethno-racial group, lifetime of homelessness education, level of needs, smoking, drug abuse or dependence, alcohol abuse or dependence, and BMI. Fit of the full model (Model 6): Prob>F: 0.001

We present findings from logistic regression in forest plots for unadjusted and variously adjusted odds ratios (95% confidence interval and *p*-value) for each outcome. The associations between the DPs and the CDs with more than 30 observations (hypertension, diabetes, asthma, chronic bronchitis/emphysema, stomach or intestinal ulcer, IBD, migraine, arthritis, kidney/bladder problem, iron-deficiency anemia, and number of CDs) (Figs. 1, 2 and 4) were adjusted for gender, age, and ethno-racial group, lifetime of homelessness, education, level of needs, smoking, drug abuse or dependence, alcohol abuse, and BMI. The associations between DPs and heart disease, stroke, thyroid problems, and liver disease (Fig. 3) were only adjusted for age, ethno-racial group and level of need for mental health services due to the few observations (< 30).

Positive statistically significant associations between having DPs and the following nine CDs were found before and after adjusting for the potential confounding variables: diabetes (Fig. 1b), chronic bronchitis/emphysema (Fig. 1d), stomach or intestinal ulcer (Fig. 1e), IBD (Fig. 1e), migraine (Fig. 2a), arthritis (Fig. 2b), kidney/bladder problems (Fig. 2c), iron-deficiency anemia (Fig. 2d), and heart disease (Fig. 3a).

Among the previous CDs, the highest magnitude of association (Adjusted odds ratio (AOR): > 3.0) was observed with heart disease, stomach or intestinal ulcer, and anemia, whilst the lowest magnitude association was that related to migraine. No statistically significant associations were found between DPs, hypertension (Fig. 1a), asthma (Fig. 1c), Stroke (Fig. 3b), thyroid problems (Fig. 3c), and liver disease (Fig. 3d). Finally, a positive and statistically significant association (incident rate ratios (IRR)) between DPs and the number of CDs was also observed (Fig. 4).

## Discussion

In our homeless population from the Toronto site of the At Home/Chez Soi trial, we found moderate positive associations (AOR: ≥ 3.5) between DPs and heart disease, stomach or intestinal ulcer, and anemia, and small-to-moderate positive associations (AOR: ≥ 2.0 and < 3.0) between DPs and diabetes, chronic bronchitis/emphysema, IBD, arthritis, and kidney/bladder problems. Less intense but statistically significant associations (AOR: ≥ 1.5 and < 2.0) were found for DPs with migraine and the number of CDs.

These positive associations may be a result of the potential exposure of our participants to risk factors shared by both DPs and those CDs such as age, low socioeconomic conditions, unhealthy lifestyles (smoking, alcohol and drug use), obesity, and poor mental health. However, we adjusted for these key variables in our analysis, and upon adjustment, no substantial changes in the magnitude of the effects were observed.

Our study findings support the hypothesised biological relationship between poor dental health and some inflammatory-based CDs such as heart diseases, chronic bronchitis/emphysema, IBD, arthritis and diabetes. For instance, a positive association between DPs and heart problems such as ischemic heart disease, heart failure, and atrial fibrillation have been reported in numerous studies in the general population [7, 40]. Related to bronchitis/emphysema, DPs such as periodontitis has been reported to be more prevalent and incident in people suffering from chronic obstructive pulmonary disease (COPD) [11, 41]. Moreover, people with COPD tend to have worse DPs manifestations (e.g., deeper periodontal pockets, gingival inflammation and bleeding) [42]. Regarding IBD, a higher prevalence of oral problems including buccal mucosa and gingiva disorders have been found in people who have Crohn’s disease and colitis [43]. Associations of reverse directionality between dental disorders with IBD have been reported as well [43]. With respect to arthritis, having a higher occurrence of DPs such as gingival problems, pockets depth, periodontitis, and poor oral hygiene index have been found among people with rheumatoid arthritis than those without [44]. Likewise, a positive association between DPs and high risk of incident palindromic-rheumatism has been reported [45]. The coexistence of DPs with diabetes and chronic kidney conditions has also been documented [46, 47].

The association observed between DPs with specific CDs might be explained by linked infective, inflammatory and immune mechanisms. The inflammatory and immune response originating from tissue damage, alteration of the local microbiota, or infections in the oral cavity and dental structures could propagate through the body, triggering the activation, deregulation, or damage of other local body tissues and physiological functions (e.g., infecting local structures, increasing the production of antigens and neutrophils and cytokines release) [48–50]. These underlying mechanisms might contribute to the development of inflammatory-based CDs. The reverse biological pathway might also be likely, where damages in other body structures trigger or exacerbate an inflammatory and immune response contributing to oral disorders including DPs. It is also possible that the observed associations are due to the poor mental health status [51] combined with the disadvantaged socioeconomic conditions, social exclusion, systemic barriers, and enduring discrimination and stigma faced by homeless people [5, 19, 25, 27, 52], which increase their likelihood of having a neglected physical health status and higher burden of chronic comorbidities, including dental diseases.

Our study also showed a positive relationship between DPs and less studied CDs such as stomach or intestinal ulcer, migraine and anemia. DPs such as periodontitis and tooth loss have been found to be associated with an increased risk of gastric and duodenal ulcer in prior literature [53, 54]. The potential presence of Helicobacter pylori in the oral cavity might be one of the explanations for our observed findings, as it is likely that due to their disadvantaged socioeconomic conditions, people experiencing chronic homelessness are more exposed to these bacteria. In fact, Helicobacter pylori infection is associated with poverty and unhygienic environments [55]. Studies have suggested that the oral cavity is also a reservoir for Helicobacter pylori, the main contributor of gastro-duodenal ulcers [56]. Indeed, Helicobacter pylori has been found simultaneously in the oral and stomach cavities [57]. Other authors have also suggested that microorganisms different to the Helicobacter pylori such as P. Gingevalis (a common bacteria associated with periodontitis) can alternate the gut microbiota and intestinal-relate functioning, inducing several local harmful responses such as inflammation or damage in the stomach and duodenum [58]. Related to migraine, our results lend support to the hypothesis that poor dental health may be a risk factor for a chronic migraine as documented in few prior studies [59], where particularly, the neurological inflammation, or endothelial, immunity and matrix protease dysfunctions can act as the potential linked pathways [60]. Concerning the association between DPs and iron-deficiency anaemia, a recent meta-analysis showed that individuals with chronic periodontitis have lower levels of hemoglobin, erythrocytes and hematocrit biomarkers [61], suggesting that the inflammatory response in chronic DPs might leading to anemia.

The positive associations between DPs and all the above discussed CDs could reflect inequalities in accessing and using dental care services and prescription drugs by individuals who are poor and socially excluded, such as homeless individuals, even within the context of universal health care systems such as in Canada [62]. Although universal health care systems are built around the principle of providing access according to health need rather than the ability to pay [62], they do not cover many of the health services needed to meet the many care needs of disadvantaged individuals [62]. For example, in Canada, the majority of dental care services are provided by private clinics and funded by private insurance or out-of-pocket payments [62, 63] and are therefore poorly accessible to low-income and socially excluded people. Moreover, Canada is the only socioeconomically developed country that provides universal coverage for health care services but not for prescription drugs [62, 64]. All of this contributes further to a high economic and comorbidity burden and health inequities for low-income people without private insurance. In other developed countries such as the United Kingdom, low-income households, people with chronic conditions, and individuals aged less 16 years or more than 60 years are exempt from paying for drugs prescriptions, as well as certain dental care services, or are entitled to receive co-payment for those services [65]. Thus, enhancing free access to dental care and prescription drugs for managing chronic comorbidities can mitigate the high burden of CDs and DPs.

In our study, we found no associations between chronic DPs and stroke, hypertension, asthma, thyroid problems and liver disease, which contrasts with some studies carried out in the general population [8, 66, 67]. It has been suggested that exposures to unhygienic conditions, overcrowding, pets, outdoor spaces, and diversity of microorganisms might have a potential protective effect for asthma and asthma symptoms in some individuals [68]. In our homeless population, these kind of exposures are indeed common. However, it is also possible that the DPs and their related inflammatory and immune responses previously discussed have no role in the pathogenesis of these diseases; or that the few events observed especially for stroke, thyroid and live problems did not allow enough statistical power for detecting significant associations.

Overall, our study findings suggest that DPs and several CDs are strongly associated in homeless adults, possibly contributing to their poor health profiles. Further, they strongly support the need for proper medical and dental management of these conditions, with primary, secondary and tertiary prevention measures, highlighting the necessity for appropriate, comprehensive and continuous medical care for this population [18, 25, 27] even within high-income countries such as Canada. Moreover, getting access to dental treatments to mitigate DPs, an area that is not well covered with public insurance in many settings, is also a priority. Better yet, dental health that is integrated into the social and health programs serving individuals experiencing homelessness such as supportive housing and social programs, outreach programs, and shelter services would be beneficial as it may ease access to these services.

In addition, structural and administrative barriers that homeless people experience, such as lack of health coverage, limited access to and provision of both preventive and treatment services for dental and overall health conditions [15–19, 25, 27], should be eliminated to reduce the premature morbimortality of this population. Furthermore, to reduce experiences of discrimination and stigma, it is essential that health care professionals, including dentists, [16, 17] receive training on serving homeless people in their settings.

Although our study is one of the few in examining the associations between DP and several CDs in the homeless population, it is not excluded from the following potential limitations that should be considered when interpreting the results. First, except for hypertension, we used self-reported CDs (lasted 6 months or more) rather than medical examination or biomarkers, which may be biased by the unawareness of our population on whether or not they were suffering from the studied conditions. Second, we did not have specific information on the type of DP (e.g., periodontitis, number of teeth, damage in the oral bone structures), which may have allowed us to assess the effect of specific DPs on the studied CDs. However, the criterion used for reporting the dental problem and CDs was that used in many survey-based health studies, which was whether a participant had experienced DPs for at least 6 months or more. This is a robust proxy for serious dental health problems rather than for relatively brief and less severe disorders. Due to the cross-sectional nature of our analysis, it was not possible to separate the temporal relationship between DP and the studied CDs. Therefore, causal inference cannot be drawn. Third, we used MI to handle the missing data in exposure, outcomes and some co-variates, which may not represent the associations that would have been observed if all data was complete. However, we carefully specified and tested the imputed models, indicating high appropriateness and efficiency in estimating accurate results. Fourth, for CDs (heart disease, stroke, thyroid problems, and liver disease) that affected a smaller sample of our population (less than 30 subjects), we only adjusted for key variables [39]. Therefore, the observed findings for those outcomes may be affected by the lack of control for other potential confounding factors. Fifth, we were unable to adjust our findings for use of dental health care, as this information was not collected. Thus, the observed associations between DPs and CDs may have differed if we were able to account for dental care utilization. Finally, our results may not be generalizable to other homeless populations outside our study setting or to different health care systems.

## Conclusions

In conclusion, dental health problems in homeless individuals are associated with several CDs. Dental care should be better integrated into existing social and health programs serving this population to improve their overall health status.

## Supplementary information

**Additional file 1: Table S1**. Univariate description of the baseline characteristics of the study participants, AH/CS Toronto Site. **Table S2.** Summary of the Imputed model performed for the study variables with missing data, AH/CS, Toronto Site. **Figure S1**. Comparison of the distribution of the observed, imputed and completed datasets in the first 10 (1 to 10/100) imputed datasets for the BMI variable. **Table S3.** Comparison of the proportions in the observed, imputed and completed dataset in the first 10 imputed datasets for the iron-deficiency anemia variable. **Table S4.** Comparison of the proportions in the observed, imputed and completed dataset in the first 10 imputed datasets for the stomach or intestinal ulcer variable. **Table S5.** Comparison of the proportions in the observed, imputed and completed dataset in the first 10 imputed datasets for the arthritis variable. **Table S6.** Comparison of the proportions in the observed, imputed and completed dataset in the first 10 imputed datasets for the thyroid problem variable. **Table S7.** Comparison of the proportions in the observed, imputed and completed dataset in the first 10 imputed datasets for the diabetes variable. **Table S8.** Comparison of the proportions in the observed, imputed and completed dataset in the first 10 imputed datasets for the liver disease (other than hepatitis) variable. **Table S9.** Comparison of the proportions in the observed, imputed and completed dataset in the first 10 imputed datasets for heart disease. **Table S10.** Comparison of the proportions in the observed, imputed and completed dataset in the first 10 imputed datasets for the kidney/bladder problems variable. **Table S11.** Comparison of the proportion in the observed, imputed and completed dataset in the first 10 imputed datasets for chronic bronchitis/emphysema variable. **Table S12.** Comparison of the proportions in the observed, imputed and completed dataset in the first 10 imputed datasets for the migraine variable. **Table S13.** Comparison of the proportions in the observed, imputed and completed dataset in the first 10 imputed datasets for the asthma variable. **Table S14.** Comparison of the proportion in the observed, imputed and completed dataset in the first 10 imputed datasets for the effect of stroke variable. **Table S15.** Comparison of the proportions in the observed, imputed and completed dataset in the first 10 imputed datasets for the dental problems variable. **Table S16.** Comparison of the proportions in the observed, imputed and completed dataset in the first 10 imputed datasets for the inflammatory bowel problems variable. **Table S17.** Comparison of the proportions in the observed, imputed and completed dataset in the first 10 imputed datasets for the hypertension variable. **Table S18.** Comparison of the proportions in the observed, imputed and completed dataset in the first 10 imputed datasets for the lifetime homelessness variable. **Table S19.** Comparison of the proportions in the observed, imputed and completed dataset in the first 10 imputed datasets for the ethno-racial group variable. **Table S20.** Comparison of the proportions in the observed, imputed and completed dataset in the first 10 imputed datasets for the education variable. **Table S21.** Comparison of the proportions in the observed, imputed and completed dataset in the first 10 imputed datasets for the smoking variable.

## Data Availability

Anonymized AH/CS participant data, study protocol, informed consent forms, survey forms, and statistical analysis plan from the AH/CS Toronto site study, as well as those related to the present paper, will be available to investigators for studies that have received approval from independent research committees or research ethics boards. Study proposals and data access requests should be sent to Dr. Stephen Hwang at Stephen.Hwang@unityhealth.to. All study proposals and data requests will be further reviewed by the AH/CS team, Toronto site. Data sharing agreements between the requestors and AH/CS principal investigators needed to be completed prior to accessing the data.

## Abbreviations

DPs: Dental problems
CDs: Chronic diseases
AOR: Adjusted odds ratios
SEP: Socioeconomic position
CVD: Cardiovascular diseases
AH/CS: Home/Chez Soi study
IBD: Inflammatory bowel disease
