# Supplementary material for: Dental problems and chronic diseases in mentally ill homeless adults: a cross-sectional study
Source: BMC Public Health. 2020 Mar 30;20:419. doi: 10.1186/s12889-020-08499-7 (PMC7106680; doi:10.1186/s12889-020-08499-7)
Supplement: Supplementary file 1 — Additional file 1: Table S1. Univariate description of the baseline characteristics of the study participants, AH/CS Toronto Site. Table S2. Summary of the Imputed model performed for the study variables with missing data, AH/CS, Toronto Site. Figure S1. Comparison of the distribution of the observed, imputed and completed datasets in the first 10 (1 to 10/100) imputed datasets for the BMI variable. Table S3. Comparison of the proportions in the observed, imputed and completed dataset in the first 10 imputed datasets for the iron-deficiency anemia variable. Table S4. Comparison of the proportions in the observed, imputed and completed dataset in the first 10 imputed datasets for the stomach or intestinal ulcer variable. Table S5. Comparison of the proportions in the observed, imputed and completed dataset in the first 10 imputed datasets for the arthritis variable. Table S6. Comparison of the proportions in the observed, imputed and completed dataset in the first 10 imputed datasets for the thyroid problem variable. Table S7. Comparison of the proportions in the observed, imputed and completed dataset in the first 10 imputed datasets for the diabetes variable. Table S8. Comparison of the proportions in the observed, imputed and completed dataset in the first 10 imputed datasets for the liver disease (other than hepatitis) variable. Table S9. Comparison of the proportions in the observed, imputed and completed dataset in the first 10 imputed datasets for heart disease. Table S10. Comparison of the proportions in the observed, imputed and completed dataset in the first 10 imputed datasets for the kidney/bladder problems variable. Table S11. Comparison of the proportion in the observed, imputed and completed dataset in the first 10 imputed datasets for chronic bronchitis/emphysema variable. Table S12. Comparison of the proportions in the observed, imputed and completed dataset in the first 10 imputed datasets for the migraine variable. Table S13. Comparison of the prop [file 12889_2020_8499_MOESM1_ESM.docx]

**Additional file 1.**

**Title: Dental problems and chronic diseases in mentally ill homeless adults: A cross-sectional study.**

**Authors:** Cilia Mejia-Lancheros, James Lachaud, Rosane Nisenbaum, Andrea Hwang, Vicky Stergiopoulos, Stephen W. Hwang, Patricia O'Campo.

**Table S1.** **Univariate description of the baseline characteristics of the study participants, AH/CS Toronto Site.**

| **Characteristics at baseline** | **N=575** | **% *or* mean (SD)** | **Missing,**  **n (% )** |
| --- | --- | --- | --- |
| **Demographics and socioeconomics** |  |  |  |
| **Gender^a^** | **575** |  |  |
| Male | 394 | 68.52 |  |
| Female | 181 | 31.48 |  |
| **Age at baseline** | **575** | 40.32(11.79) |  |
| **Ethno-racial group^b^** | **550** |  | 25(4.35) |
| White | 194 | 35.27 |  |
| Black | 190 | 34.55 |  |
| Other | 166 | 30.18 |  |
| **Education** | **550** |  | 25(4.35) |
| Attended some middle/high school | 262 | 47.64 |  |
| Completed high school | 104 | 18.91 |  |
| Attended/completed college, trade  school, or university | 184 | 33.45 |  |
| **Lifetime length of homelessness** | **538** |  | 37(6.43) |
| ≤3 years | 250 | 46.47 |  |
| >3 years | 288 | 53.53 |  |
| **Risk factors** |  |  |  |
| **Smoking at baseline** | **548** |  | 27(4.70) |
| No | 149 | 27.19 |  |
| Yes(Daily or Occasionally) | 399 | 72.81 |  |
| **Drug abuse or dependence** | **575** |  |  |
| No | 307 | 53.39 |  |
| Yes | 268 | 46.61 |  |
| **Alcohol abuse or dependence** | **575** |  |  |
| No | 331 | 57.57 |  |
| Yes | 244 | 42.43 |  |
| **BMI (kg/m^2^)** | **472** | 26.50(6.12) | 103(17.91) |
| **Level of needs for mental and social services** | **575** |  |  |
| Moderate needs | 378 | 65.74 |  |
| High needs | 197 | 34.26 |  |
| **Chronic Diseases** |  |  |  |
| **Dental problems** | **546** |  | 29(5.04) |
| No | 314 | 57.51 |  |
| Yes | 232 | 42.49 |  |
| **Heart Disease** | **534** |  | 41(7.13) |
| No | 507 | 94.94 |  |
| Yes | 27 | 5.06 |  |
| **Effect of stroke** | **544** |  |  |
| No | 522 | 95.96 | 31(5.39) |
| Yes | 22 | 4.04 |  |
| **Hypertension** | **548** |  | 27(4.70) |
| No | 322 | 58.76 |  |
| Yes | 226 | 41.24 |  |
| **Diabetes** | **532** |  | 43(7.48) |
| No | 479 | 90.04 |  |
| Yes | 53 | 9.96 |  |
| **Asthma** | **543** |  | 32(5.57) |
| No | 433 | 79.74 |  |
| Yes | 110 | 20.26 |  |
| **Chronic bronchitis/emphysema** | **541** |  | 34(5.91) |
| No | 479 | 88.54 |  |
| Yes | 62 | 11.46 |  |
| **Stomach or intestinal ulcer** | **527** |  | 48(8.35) |
| No | 472 | 89.56 |  |
| Yes | 55 | 10.44 |  |
| **Inflammatory bowel disease (Crohn's disease, colitis)** | **547** |  | 28(4.87) |
| No | 486 | 88.85 |  |
| Yes | 61 | 11.15 |  |
| **Migraine^d^** | **543** |  | 32(5.57) |
| No | 381 | 70.17 |  |
| Yes | 162 | 29.83 |  |
| **Thyroid problems** | **530** |  | 45(6.26) |
| No | 511 | 96.42 |  |
| Yes | 19 | 3.58 |  |
| **Arthritis^d^** | **528** |  | 47(8.17) |
| No | 398 | 75.38 |  |
| Yes | 130 | 24.62 |  |
| **Kidney/bladder problem** | **539** |  | 36(1.91) |
| No | 482 | 89.42 |  |
| Yes | 57 | 10.58 |  |
| **Liver disease (other than hepatitis)** | **533** |  | 42(7.30) |
| No | 515 | 96.62 |  |
| Yes | 18 | 3.38 |  |
| **Iron deficiency Anemia** | **513** |  | 62(10.78) |
| No | 422 | 82.26 |  |
| Yes | 91 | 17.74 |  |
| **Number of chronic diseases (range 0 to 11)^c^** | **550** | 1.99(1.88) | 25(4.35) |

1. Female includes individuals identified as female, transsexual (n=1) or transgender (n=9) at AH/C-S baseline.
2. The black group includes African-black, Caribbean region-black, and Canadian-black. The white group includes European-white and Canadian-white. Other ethno-racial group includes Indigenous, East Asian, South Asian, South-Eastern Asian, Latin American, Indians, and Caribbean, Middle Eastern, mixed.
3. It refers to the number of CDs (heart diseases, stroke, hypertension, diabetes, asthma, bronchitis/emphysema, stomach or intestinal ulcers, inflammatory bowel disease, migraine, thyroid problem, arthritis, kidney/bladder problem, liver disease, and Iron deficiency Anemia).

**Table S2. Summary of the Imputed model performed for the study variables with missing data, AH/CS, Toronto Site.**

| **Method** |  | Multiple imputation, by chained equations (Stata version 15) | | | |
| --- | --- | --- | --- | --- | --- |
| **Number of observations** |  | 575 | | | |
| **Number of imputations** |  | 100 | | | |
| **Interactions** |  | 10000 | | | |
| **Burn-in** |  | 100 | | | |
| **Variable** | **Statistical method used** | **Complete** | **Incomplete** | **Imputed** | **Total** |
| **BMI at baseline** | Predictive mean matching (Type 2, knn: 10) | 472 | 103 | 103 | 575 |
| **Iron deficiency anemia** | Logistic regression | 513 | 62 | 62 | 575 |
| **Stomach or intestinal ulcer** | Logistic regression | 527 | 48 | 48 | 575 |
| **Arthritis** | Logistic regression | 528 | 47 | 47 | 575 |
| **Thyroid problems** | Logistic regression | 530 | 45 | 45 | 575 |
| **Diabetes** | Logistic regression | 532 | 43 | 43 | 575 |
| **Liver disease (other than hepatitis)** | Logistic regression | 533 | 42 | 42 | 575 |
| **Heart disease** | Logistic regression | 534 | 41 | 41 | 575 |
| **Lifetime homelessness** | Logistic regression | 538 | 37 | 37 | 575 |
| **Kidney/bladder problems** | Logistic regression | 539 | 36 | 36 | 575 |
| **Chronic bronchitis/emphysema** | Logistic regression | 541 | 34 | 34 | 575 |
| **Migraine** | Logistic regression | 543 | 32 | 32 | 575 |
| **Asthma** | Logistic regression | 543 | 32 | 32 | 575 |
| **Effect of stroke** | Logistic regression | 544 | 31 | 31 | 575 |
| **Dental problems** | Logistic regression | 546 | 29 | 29 | 575 |
| **Inflammatory bowel disease** | Logistic regression | 547 | 28 | 28 | 575 |
| **Hypertension** | Logistic regression | 548 | 27 | 27 | 575 |
| **Smoking** | Logistic regression | 548 | 27 | 27 | 575 |
| **Ethno-racial identity** | Multinomial logistic regression | 550 | 25 | 25 | 575 |
| **Education level** | Ordered logistic regression | 550 | 25 | 25 | 575 |

**Multiple-imputations post-estimation assessment**

**Figure S1. Comparison of the distribution of the observed, imputed and completed datasets in the first 10 (1 to 10/100) imputed datasets for the BMI variable.**

**Table S3. Comparison of the proportions in the observed, imputed and completed dataset in the first 10 imputed datasets for the iron-deficiency anemia variable.**

| **Proportions of the selected imputed variable** | | | | |
| --- | --- | --- | --- | --- |
| **First 10 Imputed datasets** | **Iron deficiency anaemia** | **Observed**  **(n=513)** | **Imputed (n=62)** | **Completed (n=575)** |
| *m=1* | No | 0.823 | 0.758 | 0.816 |
|  | Yes | 0.177 | 0.242 | 0.184 |
| *m=2* | No | 0.823 | 0.790 | 0.819 |
|  | Yes | 0.177 | 0.210 | 0.181 |
| *m=3* | No | 0.823 | 0.806 | 0.821 |
|  | Yes | 0.177 | 0.194 | 0.179 |
| *m=4* | No | 0.823 | 0.806 | 0.821 |
|  | Yes | 0.177 | 0.194 | 0.179 |
| *m=5* | No | 0.823 | 0.839 | 0.824 |
|  | Yes | 0.177 | 0.161 | 0.176 |
| *m=6* | No | 0.823 | 0.774 | 0.817 |
|  | Yes | 0.177 | 0.226 | 0.183 |
| *m=7* | No | 0.823 | 0.758 | 0.816 |
|  | Yes | 0.177 | 0.242 | 0.184 |
| *m=8* | No | 0.823 | 0.903 | 0.831 |
|  | Yes | 0.177 | 0.097 | 0.169 |
| *m=9* | No | 0.823 | 0.903 | 0.831 |
|  | Yes | 0.177 | 0.097 | 0.169 |
| *m=10* | No | 0.823 | 0.839 | 0.824 |
|  | Yes | 0.177 | 0.161 | 0.176 |

**Table S4. Comparison of the proportions in the observed, imputed and completed dataset in the first 10 imputed datasets for the stomach or intestinal ulcer variable.**

| **Proportions of the selected imputed variable** | | | | |
| --- | --- | --- | --- | --- |
| **First 10 Imputed datasets** | **Stomach or intestinal ulcer** | **Observed**  **(n=527)** | **Imputed (n=48)** | **Completed (n=575)** |
| *m=1* | No | 0.896 | 0.875 | 0.894 |
|  | Yes | 0.104 | 0.125 | 0.106 |
| *m=2* | No | 0.896 | 0.792 | 0.887 |
|  | Yes | 0.104 | 0.208 | 0.113 |
| *m=3* | No | 0.896 | 0.854 | 0.892 |
|  | Yes | 0.104 | 0.146 | 0.108 |
| *m=4* | No | 0.896 | 0.833 | 0.890 |
|  | Yes | 0.104 | 0.167 | 0.110 |
| *m=5* | No | 0.896 | 0.875 | 0.894 |
|  | Yes | 0.104 | 0.125 | 0.106 |
| *m=6* | No | 0.896 | 0.750 | 0.883 |
|  | Yes | 0.104 | 0.250 | 0.117 |
| *m=7* | No | 0.896 | 0.854 | 0.892 |
|  | Yes | 0.104 | 0.146 | 0.108 |
| *m=8* | No | 0.896 | 0.854 | 0.892 |
|  | Yes | 0.104 | 0.146 | 0.108 |
| *m=9* | No | 0.896 | 0.792 | 0.887 |
|  | Yes | 0.104 | 0.208 | 0.113 |
| *m=10* | No | 0.896 | 0.688 | 0.878 |
|  | Yes | 0.104 | 0.313 | 0.122 |

**Table S5. Comparison of the proportions in the observed, imputed and completed dataset in the first 10 imputed datasets for the arthritis variable.**

| **Proportions of the selected imputed variable** | | | | |
| --- | --- | --- | --- | --- |
| **First 10 Imputed datasets** | **Arthritis** | **Observed**  **(n=528)** | **Imputed (n=47)** | **Completed (n=575)** |
| *m=1* | No | 0.754 | 0.574 | 0.739 |
|  | Yes | 0.246 | 0.426 | 0.261 |
| *m=2* | No | 0.754 | 0.723 | 0.751 |
|  | Yes | 0.246 | 0.277 | 0.249 |
| *m=3* | No | 0.754 | 0.532 | 0.736 |
|  | Yes | 0.246 | 0.468 | 0.264 |
| *m=4* | No | 0.754 | 0.787 | 0.757 |
|  | Yes | 0.246 | 0.213 | 0.243 |
| *m=5* | No | 0.754 | 0.702 | 0.750 |
|  | Yes | 0.246 | 0.298 | 0.250 |
| *m=6* | No | 0.754 | 0.745 | 0.753 |
|  | Yes | 0.246 | 0.255 | 0.247 |
| *m=7* | No | 0.754 | 0.702 | 0.750 |
|  | Yes | 0.246 | 0.298 | 0.250 |
| *m=8* | No | 0.754 | 0.745 | 0.753 |
|  | Yes | 0.246 | 0.255 | 0.247 |
| *m=9* | No | 0.754 | 0.702 | 0.750 |
|  | Yes | 0.246 | 0.298 | 0.250 |
| *m=10* | No | 0.754 | 0.596 | 0.741 |
|  | Yes | 0.246 | 0.404 | 0.259 |

**Table S6. Comparison of the proportions in the observed, imputed and completed dataset in the first 10 imputed datasets for the thyroid problem variable.**

| **Proportions of the selected imputed variable** | | | | |
| --- | --- | --- | --- | --- |
| **First 10 Imputed datasets** | **Thyroid problem** | **Observed**  **(n=530)** | **Imputed (n=45)** | **Completed (n=575)** |
| *m=1* | No | 0.964 | 0.889 | 0.958 |
|  | Yes | 0.036 | 0.111 | 0.042 |
| *m=2* | No | 0.964 | 0.956 | 0.963 |
|  | Yes | 0.036 | 0.044 | 0.037 |
| *m=3* | No | 0.964 | 0.844 | 0.955 |
|  | Yes | 0.036 | 0.156 | 0.045 |
| *m=4* | No | 0.964 | 0.867 | 0.957 |
|  | Yes | 0.036 | 0.133 | 0.043 |
| *m=5* | No | 0.964 | 0.956 | 0.963 |
|  | Yes | 0.036 | 0.044 | 0.037 |
| *m=6* | No | 0.964 | 0.844 | 0.955 |
|  | Yes | 0.036 | 0.156 | 0.045 |
| *m=7* | No | 0.964 | 0.956 | 0.963 |
|  | Yes | 0.036 | 0.044 | 0.037 |
| *m=8* | No | 0.964 | 0.956 | 0.963 |
|  | Yes | 0.036 | 0.044 | 0.037 |
| *m=9* | No | 0.964 | 0.889 | 0.958 |
|  | Yes | 0.036 | 0.111 | 0.042 |
| *m=10* | No | 0.964 | 0.956 | 0.963 |
|  | Yes | 0.036 | 0.044 | 0.037 |

**Table S7. Comparison of the proportions in the observed, imputed and completed dataset in the first 10 imputed datasets for the diabetes variable.**

| **Proportions of the selected imputed variable** | | | | |
| --- | --- | --- | --- | --- |
| **First 10 Imputed datasets** | **Thyroid problem** | **Observed**  **(n=532)** | **Imputed (n=43)** | **Completed (n=575)** |
| *m=1* | No | 0.900 | 0.814 | 0.894 |
|  | Yes | 0.100 | 0.186 | 0.106 |
| *m=2* | No | 0.900 | 0.698 | 0.885 |
|  | Yes | 0.100 | 0.302 | 0.115 |
| *m=3* | No | 0.900 | 0.791 | 0.892 |
|  | Yes | 0.100 | 0.209 | 0.108 |
| *m=4* | No | 0.900 | 0.674 | 0.883 |
|  | Yes | 0.100 | 0.326 | 0.117 |
| *m=5* | No | 0.900 | 0.791 | 0.892 |
|  | Yes | 0.100 | 0.209 | 0.108 |
| *m=6* | No | 0.900 | 0.837 | 0.896 |
|  | Yes | 0.100 | 0.163 | 0.104 |
| *m=7* | No | 0.900 | 0.860 | 0.897 |
|  | Yes | 0.100 | 0.140 | 0.103 |
| *m=8* | No | 0.900 | 0.953 | 0.904 |
|  | Yes | 0.100 | 0.047 | 0.096 |
| *m=9* | No | 0.900 | 0.698 | 0.885 |
|  | Yes | 0.100 | 0.302 | 0.115 |
| *m=10* | No | 0.900 | 0.744 | 0.889 |
|  | Yes | 0.100 | 0.256 | 0.104 |

**Table S8. Comparison of the proportions in the observed, imputed and completed dataset in the first 10 imputed datasets for the liver disease (other than hepatitis) variable.**

| **Proportions of the selected imputed variable** | | | | |
| --- | --- | --- | --- | --- |
| **Imputed dataset** | **Liver disease** | **Observed**  **(n=533)** | **Imputed (n=42)** | **Completed (n=575)** |
| *m=1* | No | 0.966 | 0.857 | 0.958 |
|  | Yes | 0.034 | 0.143 | 0.042 |
| *m=2* | No | 0.966 | 0.881 | 0.960 |
|  | Yes | 0.034 | 0.119 | 0.040 |
| *m=3* | No | 0.966 | 0.881 | 0.960 |
|  | Yes | 0.034 | 0.119 | 0.040 |
| *m=4* | No | 0.966 | 0.929 | 0.963 |
|  | Yes | 0.034 | 0.071 | 0.037 |
| *m=5* | No | 0.966 | 0.952 | 0.965 |
|  | Yes | 0.034 | 0.048 | 0.035 |
| *m=6* | No | 0.966 | 0.905 | 0.962 |
|  | Yes | 0.034 | 0.095 | 0.038 |
| *m=7* | No | 0.966 | 0.905 | 0.962 |
|  | Yes | 0.034 | 0.095 | 0.038 |
| *m=8* | No | 0.966 | 0.833 | 0.957 |
|  | Yes | 0.034 | 0.167 | 0.043 |
| *m=9* | No | 0.966 | 0.786 | 0.953 |
|  | Yes | 0.034 | 0.214 | 0.047 |
| *m=10* | No | 0.966 | 0.905 | 0.962 |
|  | Yes | 0.034 | 0.095 | 0.038 |

**Table S9. Comparison of the proportions in the observed, imputed and completed dataset in the first 10 imputed datasets for heart disease.**

| **Proportions of the selected imputed variable** | | | | |
| --- | --- | --- | --- | --- |
| **Imputed dataset** | **Heart disease** | **Observed**  **(n=534)** | **Imputed (n=41)** | **Completed (n=575)** |
| *m=1* | No | 0.949 | 0.878 | 0.944 |
|  | Yes | 0.051 | 0.122 | 0.056 |
| *m=2* | No | 0.949 | 0.805 | 0.939 |
|  | Yes | 0.051 | 0.195 | 0.061 |
| *m=3* | No | 0.949 | 0.780 | 0.937 |
|  | Yes | 0.051 | 0.220 | 0.063 |
| *m=4* | No | 0.949 | 0.829 | 0.941 |
|  | Yes | 0.051 | 0.171 | 0.059 |
| *m=5* | No | 0.949 | 0.927 | 0.948 |
|  | Yes | 0.051 | 0.073 | 0.052 |
| *m=6* | No | 0.949 | 0.902 | 0.946 |
|  | Yes | 0.949 | 0.902 | 0.946 |
| *m=7* | No | 0.949 | 0.927 | 0.948 |
|  | Yes | 0.051 | 0.073 | 0.052 |
| *m=8* | No | 0.949 | 0.878 | 0.944 |
|  | Yes | 0.051 | 0.122 | 0.056 |
| *m=9* | No | 0.949 | 0.829 | 0.941 |
|  | Yes | 0.051 | 0.171 | 0.059 |
| *m=10* | No | 0.949 | 0.854 | 0.943 |
|  | Yes | 0.051 | 0.146 | 0.057 |

**Table S10. Comparison of the proportions in the observed, imputed and completed dataset in the first 10 imputed datasets for the kidney/bladder problems variable.**

| **Proportions of the selected imputed variable** | | | | |
| --- | --- | --- | --- | --- |
| **Imputed dataset** | **Kidney/bladder problems** | **Observed**  **(n=539)** | **Imputed (n=36)** | **Completed (n=575)** |
| *m=1* | No | 0.894 | 0.833 | 0.890 |
|  | Yes | 0.106 | 0.167 | 0.110 |
| *m=2* | No | 0.894 | 0.833 | 0.890 |
|  | Yes | 0.106 | 0.167 | 0.110 |
| *m=3* | No | 0.894 | 0.806 | 0.889 |
|  | Yes | 0.106 | 0.194 | 0.111 |
| *m=4* | No | 0.894 | 0.889 | 0.894 |
|  | Yes | 0.106 | 0.111 | 0.106 |
| *m=5* | No | 0.894 | 0.972 | 0.899 |
|  | Yes | 0.106 | 0.028 | 0.101 |
| *m=6* | No | 0.894 | 0.778 | 0.887 |
|  | Yes | 0.106 | 0.222 | 0.113 |
| *m=7* | No | 0.894 | 0.889 | 0.894 |
|  | Yes | 0.106 | 0.111 | 0.106 |
| *m=8* | No | 0.894 | 0.889 | 0.894 |
|  | Yes | 0.106 | 0.111 | 0.106 |
| *m=9* | No | 0.894 | 0.806 | 0.889 |
|  | Yes | 0.106 | 0.194 | 0.111 |
| *m=10* | No | 0.894 | 0.806 | 0.889 |
|  | Yes | 0.106 | 0.194 | 0.111 |

**Table S11. Comparison of the proportion in the observed, imputed and completed dataset in the first 10 imputed datasets for chronic bronchitis/emphysema variable.**

| **Proportions of the selected imputed variable** | | | | |
| --- | --- | --- | --- | --- |
| **Imputed dataset** | **Chronic bronchitis/emphysema** | **Observed**  **(n= 541)** | **Imputed (n=34)** | **Completed (n=575)** |
| *m=1* | No | 0.885 | 0.735 | 0.877 |
|  | Yes | 0.115 | 0.265 | 0.123 |
| *m=2* | No | 0.885 | 0.794 | 0.880 |
|  | Yes | 0.115 | 0.206 | 0.120 |
| *m=3* | No | 0.885 | 0.882 | 0.885 |
|  | Yes | 0.115 | 0.118 | 0.115 |
| *m=4* | No | 0.885 | 0.794 | 0.880 |
|  | Yes | 0.115 | 0.206 | 0.120 |
| *m=5* | No | 0.885 | 0.794 | 0.880 |
|  | Yes | 0.115 | 0.206 | 0.120 |
| *m=6* | No | 0.885 | 0.853 | 0.883 |
|  | Yes | 0.115 | 0.147 | 0.117 |
| *m=7* | No | 0.885 | 0.853 | 0.883 |
|  | Yes | 0.115 | 0.147 | 0.117 |
| *m=8* | No | 0.885 | 0.824 | 0.882 |
|  | Yes | 0.115 | 0.176 | 0.118 |
| *m=9* | No | 0.885 | 0.647 | 0.871 |
|  | Yes | 0.115 | 0.353 | 0.129 |
| *m=10* | No | 0.885 | 0.824 | 0.882 |
|  | Yes | 0.115 | 0.176 | 0.118 |

**Table S12. Comparison of the proportions in the observed, imputed and completed dataset in the first 10 imputed datasets for the migraine variable.**

| **Proportions of the selected imputed variable** | | | | |
| --- | --- | --- | --- | --- |
| **Imputed dataset** | **Migraine** | **Observed**  **(n=543)** | **Imputed (n=32)** | **Completed (n=575)** |
| *m=1* | No | 0.702 | 0.688 | 0.701 |
|  | Yes | 0.298 | 0.313 | 0.299 |
| *m=2* | No | 0.702 | 0.656 | 0.699 |
|  | Yes | 0.298 | 0.344 | 0.301 |
| *m=3* | No | 0.702 | 0.719 | 0.703 |
|  | Yes | 0.297 | 0.297 | 0.297 |
| *m=4* | No | 0.702 | 0.750 | 0.704 |
|  | Yes | 0.298 | 0.250 | 0.296 |
| *m=5* | No | 0.702 | 0.844 | 0.710 |
|  | Yes | 0.298 | 0.156 | 0.290 |
| *m=6* | No | 0.702 | 0.813 | 0.708 |
|  | Yes | 0.298 | 0.188 | 0.292 |
| *m=7* | No | 0.702 | 0.813 | 0.708 |
|  | Yes | 0.298 | 0.188 | 0.292 |
| *m=8* | No | 0.702 | 0.563 | 0.708 |
|  | Yes | 0.298 | 0.188 | 0.292 |
| *m=9* | No | 0.702 | 0.625 | 0.697 |
|  | Yes | 0.298 | 0.375 | 0.303 |
| *m=10* | No | 0.702 | 0.719 | 0.703 |
|  | Yes | 0.298 | 0.281 | 0.297 |

**Table S13. Comparison of the proportions in the observed, imputed and completed dataset in the first 10 imputed datasets for the asthma variable.**

| **Proportions of the selected imputed variable** | | | | |
| --- | --- | --- | --- | --- |
| **Imputed dataset** | **Asthma** | **Observed**  **(n=543)** | **Imputed (n=32)** | **Completed (n=575)** |
| *m=1* | No | 0.797 | 0.781 | 0.797 |
|  | Yes | 0.203 | 0.219 | 0.203 |
| *m=2* | No | 0.797 | 0.875 | 0.802 |
|  | Yes | 0.203 | 0.125 | 0.198 |
| *m=3* | No | 0.797 | 0.750 | 0.795 |
|  | Yes | 0.203 | 0.250 | 0.205 |
| *m=4* | No | 0.797 | 0.750 | 0.795 |
|  | Yes | 0.203 | 0.250 | 0.205 |
| *m=5* | No | 0.797 | 0.906 | 0.803 |
|  | Yes | 0.203 | 0.094 | 0.197 |
| *m=6* | No | 0.797 | 0.813 | 0.798 |
|  | Yes | 0.203 | 0.188 | 0.202 |
| *m=7* | No | 0.797 | 0.813 | 0.798 |
|  | Yes | 0.203 | 0.188 | 0.202 |
| *m=8* | No | 0.797 | 0.750 | 0.795 |
|  | Yes | 0.203 | 0.250 | 0.205 |
| *m=9* | No | 0.797 | 0.688 | 0.791 |
|  | Yes | 0.203 | 0.313 | 0.209 |
| *m=10* | No | 0.797 | 0.656 | 0.790 |
|  | Yes | 0.203 | 0.344 | 0.210 |

**Table S14. Comparison of the proportion in the observed, imputed and completed dataset in the first 10 imputed datasets for the effect of stroke variable.**

| **Proportions of the selected imputed variable** | | | | |
| --- | --- | --- | --- | --- |
| **Imputed dataset** | **Effect of stroke** | **Observed**  **(n=544)** | **Imputed (n=31)** | **Completed (n=575)** |
| *m=1* | No | 0.960 | 0.968 | 0.960 |
|  | Yes | 0.040 | 0.032 | 0.040 |
| *m=2* | No | 0.960 | 0.871 | 0.955 |
|  | Yes | 0.040 | 0.129 | 0.045 |
| *m=3* | No | 0.960 | 0.935 | 0.958 |
|  | Yes | 0.040 | 0.065 | 0.042 |
| *m=4* | No | 0.960 | 0.806 | 0.951 |
|  | Yes | 0.040 | 0.194 | 0.049 |
| *m=5* | No | 0.960 | 0.806 | 0.951 |
|  | Yes | 0.040 | 0.194 | 0.049 |
| *m=6* | No | 0.960 | 0.935 | 0.958 |
|  | Yes | 0.040 | 0.065 | 0.042 |
| *m=7* | No | 0.960 | 0.806 | 0.951 |
|  | Yes | 0.040 | 0.194 | 0.049 |
| *m=8* | No | 0.960 | 0.935 | 0.958 |
|  | Yes | 0.040 | 0.065 | 0.042 |
| *m=9* | No | 0.960 | 0.839 | 0.953 |
|  | Yes | 0.040 | 0.161 | 0.047 |
| *m=10* | No | 0.960 | 0.871 | 0.955 |
|  | Yes | 0.040 | 0.129 | 0.045 |

**Table S15. Comparison of the proportions in the observed, imputed and completed dataset in the first 10 imputed datasets for the dental problems variable.**

| **Proportions of the selected imputed variable** | | | | |
| --- | --- | --- | --- | --- |
| **Imputed dataset** | **Dental problems** | **Observed**  **(n=546)** | **Imputed (n=29)** | **Completed (n=575)** |
| *m=1* | No | 0.575 | 0.690 | 0.581 |
|  | Yes | 0.425 | 0.310 | 0.419 |
| *m=2* | No | 0.575 | 0.621 | 0.577 |
|  | Yes | 0.425 | 0.379 | 0.423 |
| *m=3* | No | 0.575 | 0.724 | 0.583 |
|  | Yes | 0.425 | 0.276 | 0.417 |
| *m=4* | No | 0.575 | 0.621 | 0.577 |
|  | Yes | 0.425 | 0.379 | 0.423 |
| *m=5* | No | 0.575 | 0.655 | 0.579 |
|  | Yes | 0.425 | 0.345 | 0.421 |
| *m=6* | No | 0.575 | 0.621 | 0.577 |
|  | Yes | 0.425 | 0.379 | 0.423 |
| *m=7* | No | 0.575 | 0.552 | 0.574 |
|  | Yes | 0.425 | 0.448 | 0.426 |
| *m=8* | No | 0.575 | 0.724 | 0.583 |
|  | Yes | 0.425 | 0.276 | 0.417 |
| *m=9* | No | 0.575 | 0.483 | 0.570 |
|  | Yes | 0.425 | 0.517 | 0.430 |
| *m=10* | No | 0.575 | 0.621 | 0.577 |
|  | Yes | 0.425 | 0.379 | 0.423 |

**Table S16. Comparison of the proportions in the observed, imputed and completed dataset in the first 10 imputed datasets for the inflammatory bowel problems variable.**

| **Proportions of the selected imputed variable** | | | | |
| --- | --- | --- | --- | --- |
| **Imputed dataset** | **Inflammatory bowel problems** | **Observed**  **(n=547)** | **Imputed (n=28)** | **Completed (n=575)** |
| *m=1* | No | 0.888 | 0.893 | 0.889 |
|  | Yes | 0.112 | 0.107 | 0.111 |
| *m=2* | No | 0.888 | 0.786 | 0.883 |
|  | Yes | 0.112 | 0.214 | 0.117 |
| *m=3* | No | 0.888 | 0.893 | 0.889 |
|  | Yes | 0.112 | 0.107 | 0.111 |
| *m=4* | No | 0.888 | 0.893 | 0.889 |
|  | Yes | 0.112 | 0.107 | 0.111 |
| *m=5* | No | 0.888 | 0.893 | 0.889 |
|  | Yes | 0.112 | 0.107 | 0.111 |
| *m=6* | No | 0.888 | 0.821 | 0.885 |
|  | Yes | 0.112 | 0.179 | 0.115 |
| *m=7* | No | 0.888 | 0.821 | 0.885 |
|  | Yes | 0.112 | 0.179 | 0.115 |
| *m=8* | No | 0.888 | 0.929 | 0.890 |
|  | Yes | 0.112 | 0.071 | 0.110 |
| *m=9* | No | 0.888 | 0.929 | 0.890 |
|  | Yes | 0.112 | 0.071 | 0.110 |
| *m=10* | No | 0.888 | 0.929 | 0.890 |
|  | Yes | 0.112 | 0.071 | 0.110 |

**Table S17. Comparison of the proportions in the observed, imputed and completed dataset in the first 10 imputed datasets for the hypertension variable.**

| **Proportions of the selected imputed variable** | | | | |
| --- | --- | --- | --- | --- |
| **Imputed dataset** | **Hypertension** | **Observed**  **(n=548)** | **Imputed (n=27)** | **Completed (n=575)** |
| *m=1* | No | 0.588 | 0.519 | 0.584 |
|  | Yes | 0.412 | 0.481 | 0.416 |
| *m=2* | No | 0.588 | 0.370 | 0.577 |
|  | Yes | 0.412 | 0.630 | 0.423 |
| *m=3* | No | 0.588 | 0.519 | 0.584 |
|  | Yes | 0.412 | 0.481 | 0.416 |
| *m=4* | No | 0.588 | 0.407 | 0.579 |
|  | Yes | 0.412 | 0.593 | 0.421 |
| *m=5* | No | 0.588 | 0.630 | 0.590 |
|  | Yes | 0.412 | 0.370 | 0.410 |
| *m=6* | No | 0.588 | 0.667 | 0.591 |
|  | Yes | 0.412 | 0.333 | 0.409 |
| *m=7* | No | 0.588 | 0.519 | 0.584 |
|  | Yes | 0.412 | 0.481 | 0.416 |
| *m=8* | No | 0.588 | 0.593 | 0.588 |
|  | Yes | 0.412 | 0.407 | 0.412 |
| *m=9* | No | 0.588 | 0.593 | 0.588 |
|  | Yes | 0.412 | 0.407 | 0.412 |
| *m=10* | No | 0.588 | 0.481 | 0.583 |
|  | Yes | 0.412 | 0.519 | 0.417 |

**Table S18. Comparison of the proportions in the observed, imputed and completed dataset in the first 10 imputed datasets for the lifetime homelessness variable.**

| **Proportions of the selected imputed variable** | | | | |
| --- | --- | --- | --- | --- |
| **Imputed dataset** | **Lifetime homelessness** | **Observed**  **(n=** **538)** | **Imputed** (**n=37)** | **Completed (n=575)** |
| *m=1* | < 3 years | 0.465 | 0.351 | 0.457 |
|  | ≥ 3years | 0.535 | 0.649 | 0.543 |
| *m=2* | < 3 years | 0.465 | 0.459 | 0.464 |
|  | ≥ 3years | 0.535 | 0.541 | 0.536 |
| *m=3* | < 3 years | 0.465 | 0.595 | 0.473 |
|  | ≥ 3years | 0.535 | 0.405 | 0.527 |
| *m=4* | < 3 years | 0.465 | 0.405 | 0.461 |
|  | ≥ 3years | 0.535 | 0.595 | 0.539 |
| *m=5* | < 3 years | 0.465 | 0.432 | 0.463 |
|  | ≥ 3years | 0.535 | 0.568 | 0.537 |
| *m=6* | < 3 years | 0.465 | 0.432 | 0.463 |
|  | ≥ 3years | 0.535 | 0.568 | 0.537 |
| *m=7* | < 3 years | 0.465 | 0.514 | 0.468 |
|  | ≥ 3years | 0.535 | 0.486 | 0.532 |
| *m=8* | < 3 years | 0.465 | 0.405 | 0.461 |
|  | ≥ 3years | 0.535 | 0.595 | 0.539 |
| *m=9* | < 3 years | 0.465 | 0.432 | 0.463 |
|  | ≥ 3years | 0.535 | 0.568 | 0.537 |
| *m=10* | < 3 years | 0.465 | 0.432 | 0.463 |
|  | ≥ 3years | 0.535 | 0.568 | 0.537 |

**Table S19. Comparison of the proportions in the observed, imputed and completed dataset in the first 10 imputed datasets for the ethno-racial group variable.**

|  | **Proportions of the selected imputed variable** | | | |
| --- | --- | --- | --- | --- |
| **Imputed dataset** | **Ethno-racial group** | **Observed**  **(n=** **550)** | **Imputed** (**n=25)** | **Completed (n=575)** |
| *m=1* | White (Canadian, European) | 0.465 | 0.351 | 0.457 |
|  | Black (Canadian, Caribbean, African) | 0.535 | 0.649 | 0.543 |
|  | Other ethno-racial groups | 0.302 | 0.400 | 0.306 |
| *m=2* | White (Canadian, European) | 0.353 | 0.200 | 0.346 |
|  | Black (Canadian, Caribbean, African) | 0.345 | 0.360 | 0.346 |
|  | Other ethno-racial groups | 0.302 | 0.440 | 0.308 |
| *m=3* | White (Canadian, European) | 0.353 | 0.240 | 0.348 |
|  | Black (Canadian, Caribbean, African) | 0.345 | 0.440 | 0.350 |
|  | Other ethno-racial groups | 0.302 | 0.320 | 0.303 |
| *m=4* | White (Canadian, European) | 0.353 | 0.320 | 0.351 |
|  | Black (Canadian, Caribbean, African) | 0.345 | 0.360 | 0.346 |
|  | Other ethno-racial groups | 0.302 | 0.320 | 0.303 |
| *m=5* | White (Canadian, European) | 0.353 | 0.400 | 0.355 |
|  | Black (Canadian, Caribbean, African) | 0.345 | 0.520 | 0.353 |
|  | Other ethno-racial groups | 0.302 | 0.080 | 0.292 |
| *m=6* | White (Canadian, European) | 0.353 | 0.280 | 0.350 |
|  | Black (Canadian, Caribbean, African) | 0.345 | 0.480 | 0.351 |
|  | Other ethno-racial groups | 0.302 | 0.240 | 0.299 |
| *m=7* | White (Canadian, European) | 0.353 | 0.280 | 0.350 |
|  | Black (Canadian, Caribbean, African) | 0.345 | 0.440 | 0.350 |
|  | Other ethno-racial groups | 0.302 | 0.280 | 0.301 |
| *m=8* | White (Canadian, European) | 0.353 | 0.320 | 0.351 |
|  | Black (Canadian, Caribbean, African) | 0.345 | 0.520 | 0.353 |
|  | Other ethno-racial groups | 0.302 | 0.160 | 0.296 |
| *m=9* | White (Canadian, European) | 0.353 | 0.320 | 0.351 |
|  | Black (Canadian, Caribbean, African) | 0.345 | 0.320 | 0.344 |
|  | Other ethno-racial groups | 0.302 | 0.360 | 0.304 |
| *m=10* | White (Canadian, European) | 0.353 | 0.360 | 0.353 |
|  | Black (Canadian, Caribbean, African) | 0.345 | 0.280 | 0.343 |
|  | Other ethno-racial groups | 0.302 | 0.360 | 0.304 |

**Table S20. Comparison of the proportions in the observed, imputed and completed dataset in the first 10 imputed datasets for the education variable.**

|  | **Proportions of the selected imputed variable** | | | |
| --- | --- | --- | --- | --- |
| **Imputed dataset** | **Education level** | **Observed**  **(n=** **550)** | **Imputed** (**n=25)** | **Completed (n=575)** |
| *m=1* | Attended some middle/high school | 0.476 | 0.400 | 0.473 |
|  | Completed high school | 0.189 | 0.200 | 0.190 |
|  | Attended/completed graduate/postgraduate | 0.335 | 0.400 | 0.337 |
| *m=2* | Attended some middle/high school | 0.476 | 0.560 | 0.480 |
|  | Completed high school | 0.189 | 0.120 | 0.186 |
|  | Attended/completed graduate/postgraduate | 0.335 | 0.320 | 0.334 |
| *m=3* | Attended some middle/high school | 0.476 | 0.440 | 0.475 |
|  | Completed high school | 0.189 | 0.120 | 0.186 |
|  | Attended/completed graduate/postgraduate | 0.335 | 0.440 | 0.339 |
| *m=4* | Attended some middle/high school | 0.476 | 0.400 | 0.473 |
|  | Completed high school | 0.189 | 0.280 | 0.193 |
|  | Attended/completed graduate/postgraduate | 0.335 | 0.320 | 0.334 |
| *m=5* | Attended some middle/high school | 0.476 | 0.360 | 0.471 |
|  | Completed high school | 0.189 | 0.240 | 0.191 |
|  | Attended/completed graduate/postgraduate | 0.335 | 0.400 | 0.337 |
| *m=6* | Attended some middle/high school | 0.476 | 0.600 | 0.482 |
|  | Completed high school | 0.189 | 0.040 | 0.183 |
|  | Attended/completed graduate/postgraduate | 0.335 | 0.360 | 0.336 |
| *m=7* | Attended some middle/high school | 0.476 | 0.400 | 0.473 |
|  | Completed high school | 0.189 | 0.200 | 0.190 |
|  | Attended/completed graduate/postgraduate | 0.335 | 0.400 | 0.337 |
| *m=8* | Attended some middle/high school | 0.476 | 0.520 | 0.478 |
|  | Completed high school | 0.189 | 0.160 | 0.188 |
|  | Attended/completed graduate/postgraduate | 0.335 | 0.320 | 0.334 |
| *m=9* | Attended some middle/high school | 0.476 | 0.360 | 0.471 |
|  | Completed high school | 0.189 | 0.280 | 0.193 |
|  | Attended/completed graduate/postgraduate | 0.335 | 0.360 | 0.336 |
| *m=10* | Attended some middle/high school | 0.476 | 0.720 | 0.487 |
|  | Completed high school | 0.189 | 0.080 | 0.184 |
|  | Attended/completed graduate/postgraduate | 0.335 | 0.200 | 0.329 |

**Table S21. Comparison of the proportions in the observed, imputed and completed dataset in the first 10 imputed datasets for the smoking variable.**

| **Proportions of the selected imputed variable** | | | | |
| --- | --- | --- | --- | --- |
| **Imputed dataset** | **Smoking** | **Observed**  **(n=548)** | **Imputed** (**n=27)** | **Completed (n=575)** |
| *m=1* | No | 0.272 | 0.296 | 0.273 |
|  | Yes | 0.728 | 0.704 | 0.727 |
| *m=2* | No | 0.272 | 0.481 | 0.282 |
|  | Yes | 0.728 | 0.519 | 0.718 |
| *m=3* | No | 0.272 | 0.259 | 0.271 |
|  | Yes | 0.728 | 0.741 | 0.729 |
| *m=4* | No | 0.272 | 0.333 | 0.275 |
|  | Yes | 0.728 | 0.667 | 0.725 |
| *m=5* | No | 0.272 | 0.407 | 0.278 |
|  | Yes | 0.728 | 0.593 | 0.722 |
| *m=6* | No | 0.272 | 0.407 | 0.278 |
|  | Yes | 0.728 | 0.593 | 0.722 |
| *m=7* | No | 0.272 | 0.370 | 0.277 |
|  | Yes | 0.728 | 0.630 | 0.723 |
| *m=8* | No | 0.272 | 0.333 | 0.275 |
|  | Yes | 0.728 | 0.667 | 0.725 |
| *m=9* | No | 0.272 | 0.259 | 0.271 |
|  | Yes | 0.728 | 0.741 | 0.729 |
| *m=10* | No | 0.272 | 0.333 | 0.275 |
|  | Yes | 0.728 | 0.667 | 0.725 |
